# Effect of four traditional Chinese medicine monomers on mechanical barrier damage and inflammation response of IPEC-J2 cells caused by soybean 7S globulin

**DOI:** 10.3389/fvets.2025.1548866

**Published:** 2025-03-07

**Authors:** Youtian Deng, Xiaoli Wu, Yingying Wan, Junliang Deng, Huidan Deng

**Affiliations:** ^1^College of Food Science, Sichuan Agriculture University, Yaan, Sichuan, China; ^2^College of Veterinary Medicine, Sichuan Agricultural University, Chengdu, China; ^3^Key Laboratory of Animal Diseases and Environmental Hazards of Sichuan Province, Sichuan Agriculture University, Chengdu, China

**Keywords:** soybean 7S globulin, curcumin, eleutheroside E, saponin B4, forsythia A, mechanical barrier, IPEC-J2 cells

## Abstract

**Introduction:**

The soybean 7S globulin is a major allergen responsible for diarrhea in weaned piglets, leading to significant economic loss in the pig breeding industry. Therefore, there is an urgent need to find effective and safe therapeutic agents to prevent and treat diarrhea caused by soybean 7S globulin. Given the global trend toward “antibiotic alternatives,” traditional Chinese medicines (TCMs) offer a promising approach for the prevention and control of animal diseases.

**Methods:**

In this study, four TCM monomers (curcumin, eleutheroside E, saponin B4, and forsythia A) were evaluated for their protective and therapeutic effects on intestinal epithelial cells (IPEC-J2) damaged by soybean 7S globulin. The CCK8 assay, western blot assay, Elisa assay, and PCR assay were used in this study.

**Results:**

The results demonstrated that curcumin at concentrations of 0.02, 0.04, and 0.08 μg/mL, eleutheroside E at 25, and 50 μg/mL, saponin B4 at 12.5, 25, and 50 μg/mL, and forsythia A at 20, and 40 μg/mL had significant ameliorative effects on cell viability, permeability, and integrity. Furthermore, the TCM monomers alleviated the inflammatory response, reduced the disruption of tight junctions, and improved the cellular mechanical barrier. These protective effects were likely mediated through the inhibition of the Rho/ROCK signaling pathway, characterized by down regulation of RhoA, ROCK1, ROCK2, and MLKC expression.

**Discussion:**

These findings suggest that the four TCM monomers have the potential to treat diarrhea of weaned piglets caused by soybean protein.

## Introduction

The increasing consumption of soybean products, due to their high nutritional value, has led to a rise in the incidence of soybean allergies (1). Soybean 7S globulin, the primary allergenic factor in soybeans, is a major cause of diarrhea in weaned piglets (2), especially in diets containing more than 20% protein (3–5). Due to the immaturity of the intestinal systems in weaned piglets, a portion of 7S globulin and its enzymatically active peptides can pass through the epithelial cell gaps of the small intestine and enter the bloodstream and lymphatic system (6). This can trigger inflammation response (7). Studies also showed that soybean 7S globulin reduces digestive enzyme activity in the intestines of weaned piglets, affects intestinal cell function and mucosal morphology, induces apoptosis in small intestinal epithelial cells, and damages the intestinal mechanical barrier (6, 8–10). Moreover, 7S globulin can lead to histamine release, which triggers delayed-type hypersensitivity reactions (types I, III, and IV) (11). These processes elicit an immune response in weaned piglets, resulting in allergic reactions.

The Rho/ROCK signaling pathway regulates cell morphology and cytoskeleton rearrangement, and influences cell proliferation, differentiation, movement, and adhesion. Consequently, it plays a crucial role in the formation of tight junction (TJ) proteins and the regulation of intracellular inflammatory responses. In a study by Yang et al. (12), intestinal epithelial cells were treated with 5 mg/mL of soybean 7S globulin along with specific inhibitors of MLCK and ROCK, respectively. The results demonstrated that these inhibitors significantly reduced the level of IL-6, IL-8, and TNF-α, while increased IL-10, Claudin-1, and ZO-1 levels, thereby improving TJ protein expression. These findings suggest that the ROCK signaling pathway is a key mediator of cell damage caused by soybean 7S globulin.

Traditional Chinese medicine (TCM) offers the advantages of low side effects and abundant natural sources. Numerous studies have shown that TCM can help maintain the integrity of intestinal epithelial cells and protect the intestinal mucosa's basic structure. Studies have been proved that curcumin, eleutheroside E, Saponin B4 and forsythia A all have anti-inflammatory and antioxidant properties. They can regulate intestinal immunity, maintain the integrity of intestinal epithelial cells and protect intestinal mucosa in mice colitis model and LPS-induced mice inflammation models (13–21). However, the effects of curcumin, eleutheroside E, Saponin B4 and forsythia A in alleviating diarrhea caused by soybean 7S globulin in weaned piglets is still unclear.

Therefore, in this study, IPEC-J2 cells were chosen to investigate the effects and mechanisms of curcumin, eleutheroside E, saponin B4, and forsythia A on the disruption of mechanical barriers and the inflammatory response in IPEC-J2 cells caused by soybean 7S globulin. The results of this study may provide new insights for the subsequent development of feed additives to prevent and control diarrhea of weaned piglets caused by soybean protein.

## Materials and methods

### Reagents and drugs

Soybean 7S globulin (85 ± 3%) was purchased from Huaibei Food lab Technology Co., Ltd. (Huaibei, China). Curcumin, eleutheroside E, saponin B4, and forsythia A (purity > 95%) were obtained from Shanghai Yuanyuan Biological Co., Ltd. (Shanghai, China). Buffers and lysates, including dimethyl sulfoxide (DMSO), phosphate-buffered saline (PBS), D-Hank's buffer, running buffer, and PMSF lysates, were procured from Beijing Solebao Technology Co., Ltd. (Shanghai, China). A 0.25% pancreatic enzyme digestion solution and CCK-8 kits were purchased from Beyotime Biotechnology (Shanghai, China).

### Cell culture

IPEC-J2 cells (Shanghai Yingwan Biotechnology Company) were cultured in DMEM/F12 medium (Gibco, Carlsbad, NM, USA) enriched with 10% fetal bovine serum (FBS, Hyclone, Australia) and 0.25% trypsin enzyme digestion solution (Beyotime) at 37°C in a humidified atmosphere with 5% CO_2_.

### Measurement of cellular activity

The effects of the four TCM monomers on the activity of IPEC-J2 cells were assessed utilizing the CCK-8 assay. The cells were evenly spread in 96-well plates, treated with soybean 7S globulin and traditional Chinese medicine monomer for 24 h according to the experimental protocol, then 10 μL of CCK8 working liquid were added to the cells without holes. Incubate for 2–4 h before testing. The details of the experimental groups are provided in Table 1.

**Table 1:** Experimental grouping design of four Chinese herbal medicine monomers.

**[-50,11.7]7735mm Treatment Group**	**Curcumin (μg/mL)**	**Eleutheroside E (μg/mL)**	**Saponin B4 (μg/mL)**	**Forsythia A (μg/mL)**
Control	0	0	0	0
Treatment 1	0.02	12.5	12.5	10
Treatment 2	0.04	25	25	20
Treatment 3	0.08	50	50	40

### Determination of cellular permeability

The transepithelial electrical resistance (TEER) was measured in triplicate for all wells using a resistance meter. Only wells with TEER measurements exceeding 500 Ω·cm^2^ were included in the experiment. Experiments were conducted on three independent occasions, and the average values were selected for analysis. Wells without cells were used as blank controls, and their values were subtracted as blank values.


$$\begin{array}{l} \text{ TEER }\Omega \cdot \text{c}{\text{m}}^{2}\;=\left(\text{measured value}-\text{blank value}\right)\;\times \text{ effective}\\ \text{ membrane area }\left(0.33\text{cm}\right) \end{array}$$

IPEC-J2 cells were inoculated into Transwell plates (Corning Incorporated, GLW, USA) and washed with pre-warmed D-Hanks buffer (Solarbio, Shanghai, China) at 37°C. The optical density (OD) value of the lower chamber was measured at 490 nm, and the fluorescein sodium (FS) concentration was calculated according to the standard curve.


$$\begin{array}{l} \text{ FS permeability }\left(\text{\%}\right)\;=\left(\text{BL side concentration }\times \;0.7\right)/\left(60\;\times \;0.2\right)\\ \;\times \;100 \end{array}$$

### Enzyme-linked immunosorbent assay (ELISA)

All relevant experiments were conducted according to the instructions provided with the ELISA kits (Jiangsu Meimian Industrial Co., Ltd.). The concentrations of the target proteins were determined using a standard curve.

### Relative quantitative real-time PCR

Total RNA were extracted according to the manufacturer's protocol. cDNA synthesis was made with RNA (1 μg) in combination with the reverse transcription kit (FOREGENE, Chengdu, China) in accordance with corresponding specifications. The gene data came from National Center for Biotechnology Information (NCBI), and Sangon (Shanghai, China) took charge of primer design and synthesis. The mRNA expression was assessed using SYBR^®^ Premix Ex TaqTMII (RR820A, Takara, China) according to corresponding specifications. All the reactions needed to be performed at 95°C for 10 min, and then at 95°C for 10 min, at 60°C for 20 s, and at 72°C for 20 s. Subsequently, melt curves were investigated to identify PCR specificity. The resulting cDNA was adopted as a template to detect mRNA expression. The comparative Ct (ΔΔCt) method was utilized to analyze the results. Primers sequences are listed in Table 2. The 2^−Δ*ΔCt*^ method was applied to quantify the expression level of the target genes.

**Table 2:** Primer sequences.

**Gene name**	**Primer sequences (5^′^-3^′^)**	**Product size (bp)**
ZO-1	(F)CCAGGGAGAGAAGTGCCAGTAGG (R)TTTGGTGGGTTTGGTGGGTTGAC	92
Claudin-1	(F)CCATCGTCAGCACCGCACTG (R)CGACACGCAGGACATCCACAG	107
Occludin	(F)TGGCTGCCTTCTGCTTCATTGC (R)GAACACCATCACACCCAGGATAGC	130
β-actin	(F)GGCACCACACCTTCTACAACGAG (R)TCATCTTCTCACGGTTGGCTTTGG	102

### Western blot analysis

After treating IPEC-J2 cells with lysis buffer (PMSF was added immediately before use), the cells were homogenized and centrifuged to collect the supernatant. Sample buffer was added to the supernatant to obtain the total protein from the IPEC-J2 cells. Protein quantification was performed following the instructions of the BCA Protein Assay Kit (Beyotime). After protein extraction, gel preparation, sample loading, electrophoresis, membrane transfer, immunoreaction, and color development were carried out to visualize the protein bands. The relative protein expression were analyzed using ImageJ software. The ZO-1 antibody (66452-1-Ig), Occludin antibody (80545-1-PBS), Claudin-1 antibody (28674-1-AP), GAPDH antibody (60004-1-Ig), RhoA antibody (10749-1-AP), ROCK1 antibody (21850-1-AP), ROCK2 antibody (21645-1-AP), MLCK antibody (21642-1-AP) were purchased from proteintech.

### Statistical analysis

The results are presented as mean ± standard deviation (SD). One-way analysis of variance (ANOVA) was conducted utilizing GraphPad Prism software (GraphPad Software, Inc.). A value of *P* < 0.05 was deemed a remarkable difference.

## Results

### Effect of four herbal monomers on cell viability in IPEC-J2 cells treated with soybean 7S globulin

**Figure 1:**
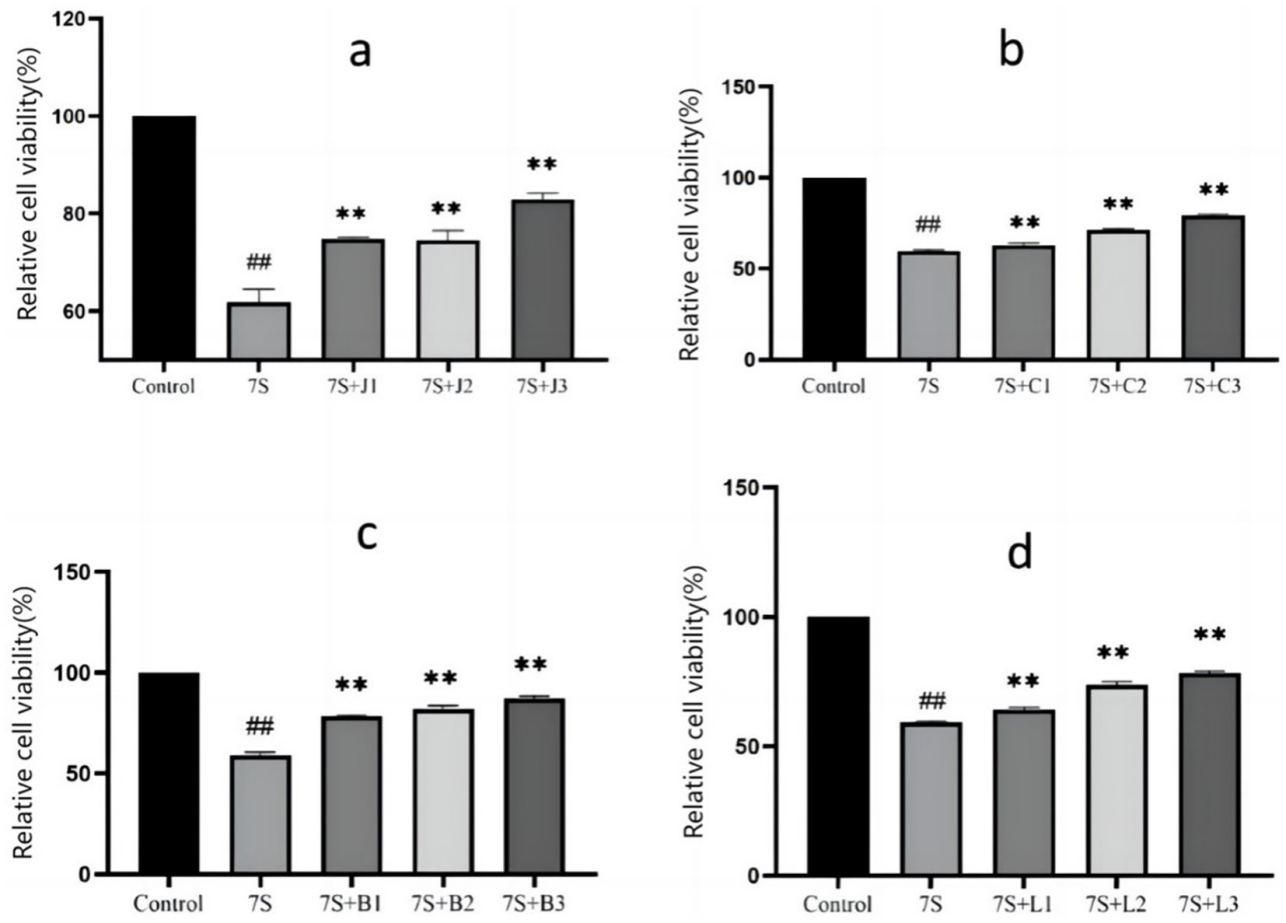
Effect of curcumin **(A)**, eleutheroside E **(B)**, saponin B4 **(C)** and forsythia A **(D)** on cells viability in IPEC-J2 cells treated with soybean 7S globulin. Control refers to the control group, while 7S refers to the 7S model group. 7S+J (1, 2, 3) means the 7S model group supplemented with 0.02, 0.04, and 0.08 μg/mL of curcumin, respectively; 7S+C (1, 2, 3) means the 7S model group supplemented with 12.5, 25, and 50 μg/mL of eleu-theroside E, respectively; 7S + B(1, 2, 3) means the 7S model group supplemented with 12.5, 25, and 50 μg/mL of saponin B4, respectively; 7S+L (1, 2, 3) means the 7S model group supplemented with 10, 20, and 40 μg/mL of forsythia A. **Indicates a highly significant difference compared with the 7S group (*P* < 0.01). ^*##*^(Compared with the control group) means *P* < 0.01.

As shown in Figure 1. When compared to the 7S group, the relative cell viability exhibited a positive linear relationship with increasing concentrations of the herbal monomers (*P* < 0.01). Curcumin at concentrations of 0.02, 0.04, and 0.08 μg/mL, eleutheroside E at 25 and 50 μg/mL, saponin B4 at 12.5, 25, and 50 μg/mL, forsythia A at 20 and 40 μg/mL were all able to mitigate the adverse effects induced by soybean 7S globulin. Among these, curcumin at 0.08 μg/mL, eleutheroside E at 50 μg/mL, saponin B4 at 50 μg/mL, and forsythia A at 40 μg/mL has the most significant protective effects.

### Effect of four TCM monomers on TEER in IPEC-J2 cells treated with soybean 7S globulin

**Figure 2:**
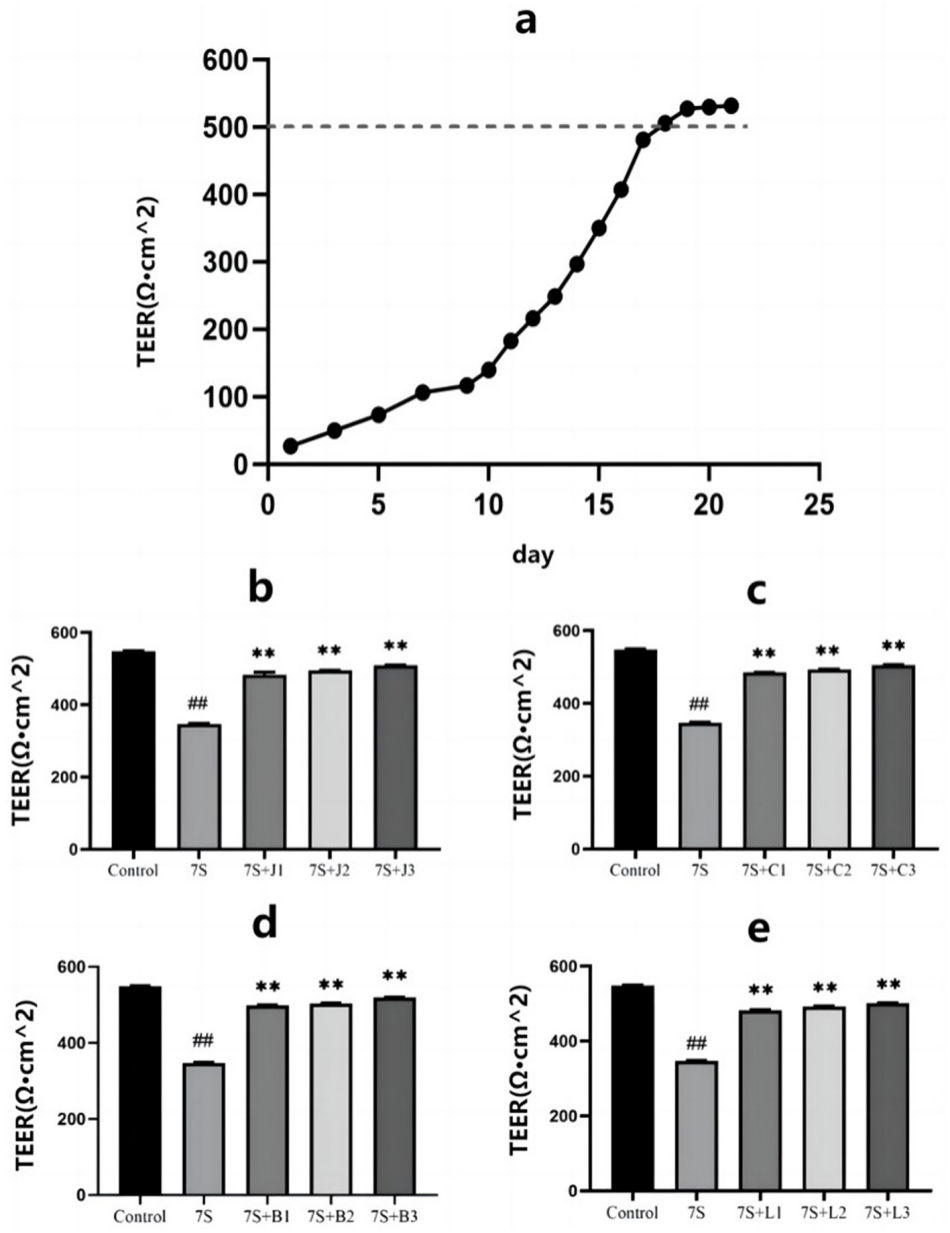
Effect of four traditional Chinese medicine monomers on the transepithelial electrical resistance in IPEC-J2 cells induced by soybean 7S globulin. **(A)** The model resistors. The TEER values of curcumin **(B)**, eleutheroside E **(C)**, saponin B4 **(D)** and forsythia A **(E)**. Data are shown as mean ± standard deviation (SD). **(Compared with the 7S group) or ^*##*^(Compared with the control group) means *P* < 0.01.

As shown in Figure 2A, the TEER of IPEC-J2 cells gradually increased over the culture period, reaching a plateau around day 18, indicating that the cells had fused into a monolayer suitable for subsequent permeability tests.

As illustrated in Figures 2B–E, treatment with curcumin (0.02, 0.04, and 0.08 μg/mL), eleutheroside E (12.5, 25, and 50 μg/mL), saponin B4 (12.5, 25, and 50 μg/mL), and forsythia A (10, 20, and 40 μg/mL) significantly increased the TEER values, indicating their protective effect on IPEC-J2 cells (*P* < 0.01). Notably, curcumin at 0.08 μg/mL, eleutheroside E at 50 μg/mL, saponin B4 at 50 μg/mL, and forsythia A at 40 μg/mL exhibited the most pronounced protective effects.

### Effect of four TCM monomers on permeability in IPEC-J2 cells treated with soybean 7S globulin

**Figure 3:**
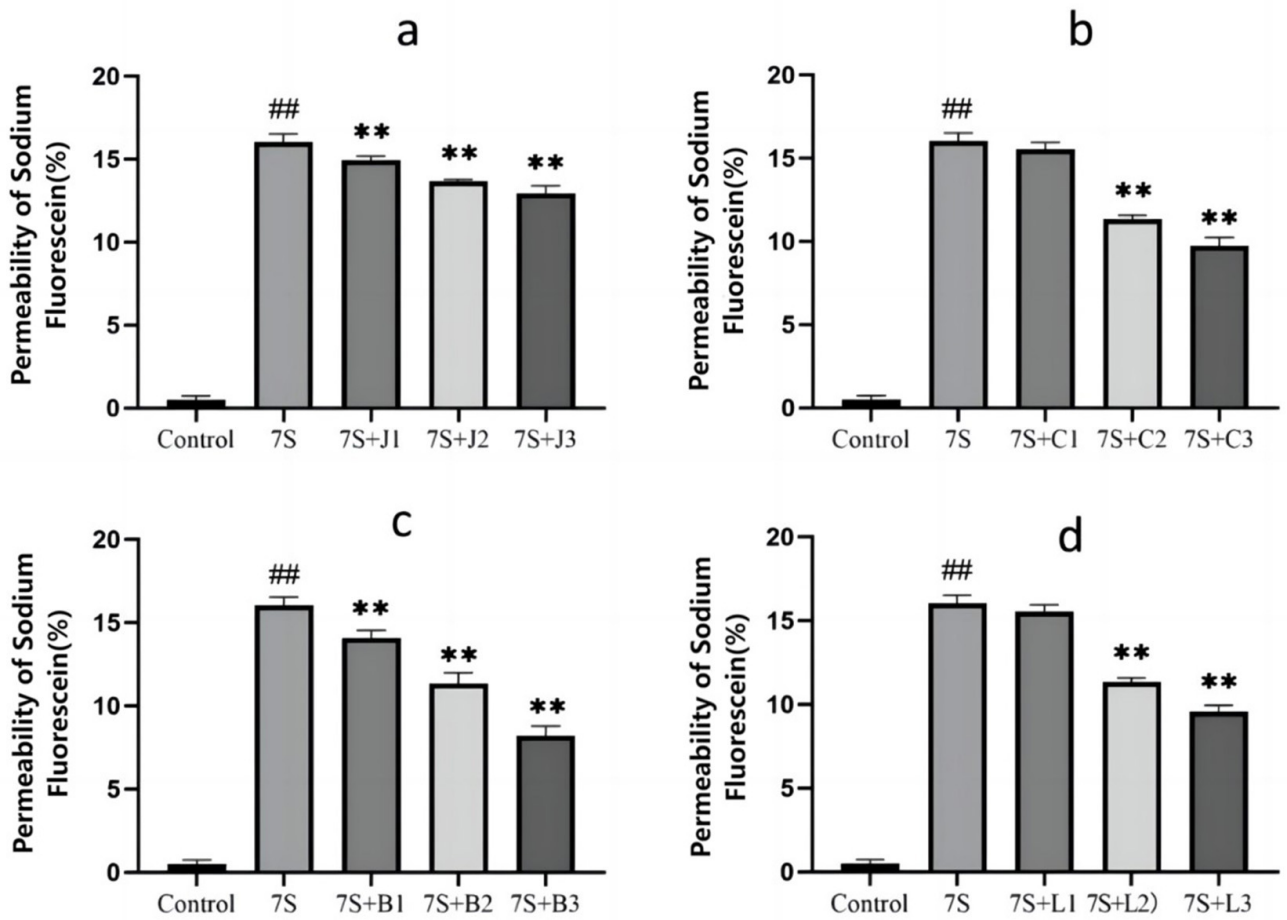
Effect of curcumin **(A)**, eleutheroside E **(B)**, saponin B4 **(C)**, and forsythia A **(D)** monomers on the permeability of fluorescein sodium in IPEC-J2 cells induced by soy-bean 7S globulin. Data are presented with the means ± standard deviation. **(Compared with the 7S group) or ^*##*^(Compared with the control group) means *P* < 0.01.

The effects of the four TCM monomers on the permeability in IPEC-J2 cells are presented in Figure 3. Compared to the 7S group, treatment with curcumin (0.02, 0.04, and 0.08 μg/mL), eleutheroside E (25 and 50 μg/mL), saponin B4 (12.5, 25, and 50 μg/mL), and forsythia A (20 and 40 μg/mL) resulted in a significant reduction in permeability (*P* < 0.01). However, no significant difference was observed for eleutheroside E at 12.5 μg/mL and forsythia A at 10 μg/mL. Among these, curcumin at 0.08 μg/mL, eleutheroside E at 50 μg/mL, saponin B4 at 50 μg/mL, and forsythia A at 40 μg/mL has the most effective reduction in permeability.

### Effect of TCM monomers on the integrity of IPEC-J2 cells treated with soybean 7S globulin

**Figure 4:**
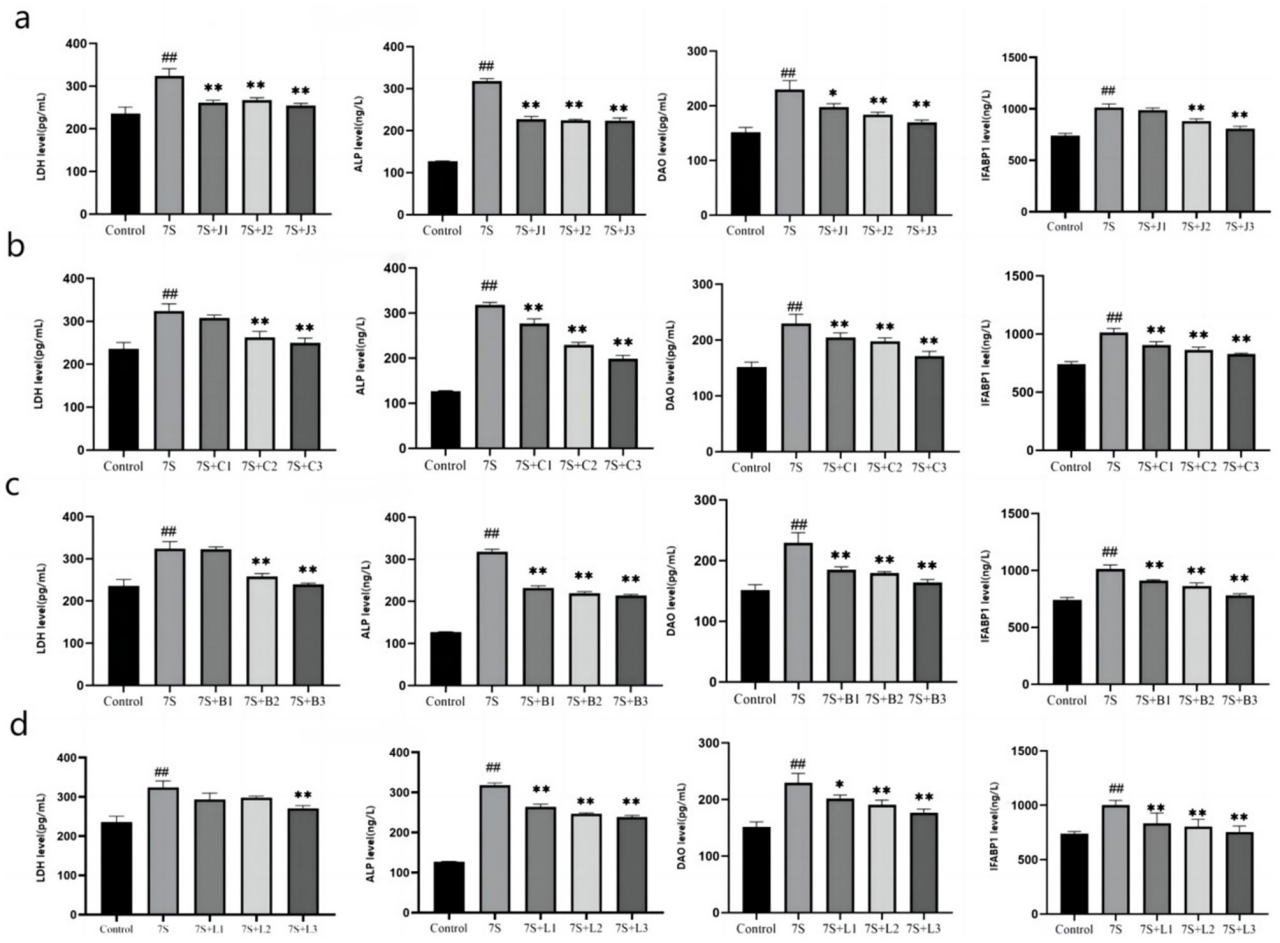
Effect of four traditional Chinese medicine monomers on the integrity of IPEC-J2 cells induced by soybean 7S globulin. Effect of curcumin **(A)**, eleutheroside E **(B)**, saponin B4 **(C)**, and forsythia A **(D)** on the LDH, ALP, DAO, and IFABP1 levels. Data are presented with the means ± standard deviation. *(Compared with the 7S group) means *P* < 0.05, **(Compared with the 7S group) or ^*##*^(Compared with the control group) means *P* < 0.01.

As shown in Figure 4, the levels of LDH, ALP, DAO, and IFABP1 were measured to assess cell integrity. Compared to the 7S group, treatment with curcumin (0.02, 0.04, and 0.08 μg/mL), eleutheroside E (25 and 50 μg/mL), saponin B4 (25 and 50 μg/mL), and forsythia A (40 μg/mL) significantly decreased these markers (*P* < 0.05 or *P* < 0.01). However, there were no significant difference observed for eleutheroside E at 12.5 μg/mL, saponin B4 at 12.5 μg/mL, and forsythia A at 10 and 20 μg/mL. Among the tested concentrations, curcumin at 0.08 μg/mL, eleutheroside E at 50 μg/mL, saponin B4 at 50 μg/mL, and forsythia A at 40 μg/mL demonstrated to has the best protective effects on cell integrity.

### Effect of four TCM monomers on inflammation factors of IPEC-J2 cells treated with soybean 7S globulin

**Figure 5:**
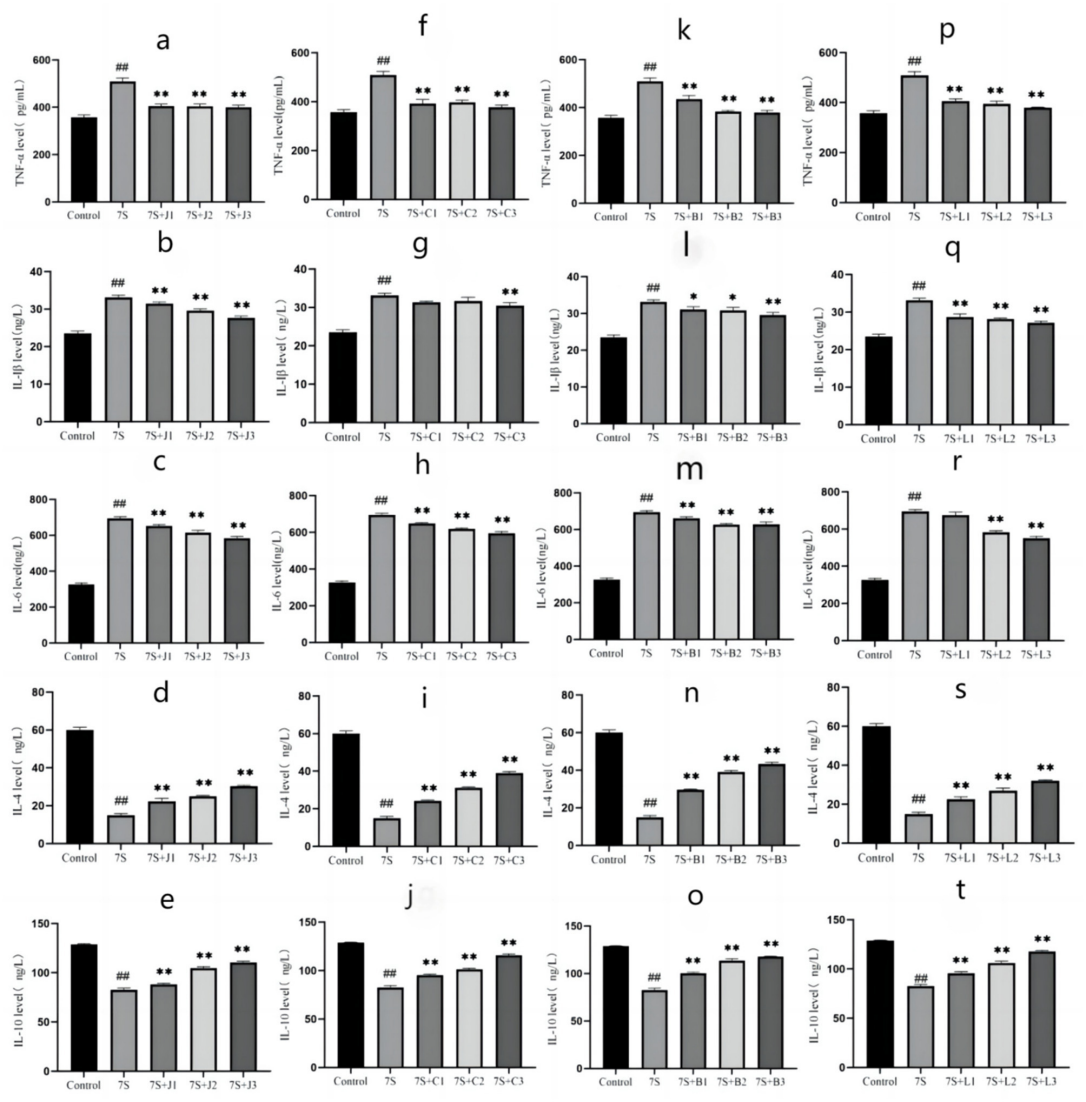
Effect of four traditional Chinese medicine monomers on IPEC-J2 cell inflammatory factors caused by soybean 7S globulin. Effect of curcumin **(A–C)**, eleutheroside E **(F–H)**, saponin B4 **(K–M)**, and forsythia A **(P–R)** on pro-inflammatory factors; Effect of curcumin **(D, E)**, eleutheroside E **(I, J)**, saponin B4 **(N, O)**, and forsythia A **(S, T)** on anti-inflammatory factors. Data are presented with the means ± standard deviation. *(Compared with the 7S group) means *P* < 0.05, **(Compared with the 7S group) or ^*##*^(Compared with the control group) means *P* < 0.01.

The effects of the four TCM monomers on inflammation related factors in IPEC-J2 cells are shown in Figure 5. Compared to the 7S group, treatment with curcumin (0.02, 0.04, and 0.08 μg/mL), eleutheroside E (12.5, 25, and 50 μg/mL), saponin B4 (12.5, 25, and 50 μg/mL), and forsythia A (10, 20, and 40 μg/mL) significantly alleviated inflammation (*P* < 0.01). The levels of pro-inflammatory cytokines TNF-α, IL-1β, and IL-6 were markedly reduced (*P* < 0.01), while the levels of anti-inflammatory cytokines IL-4 and IL-10 were notably increased (*P* < 0.01). Among these, curcumin at 0.08 μg/mL, eleutheroside E at 50 μg/mL, saponin B4 at 50 μg/mL, and forsythia A at 40 μg/mL exhibited the most pronounced anti-inflammatory effects.

### Effects of four TCM monomers on TJ mRNA and protein expression in IPEC-J2 cells treated with soybean 7S globulin

**Figure 6:**
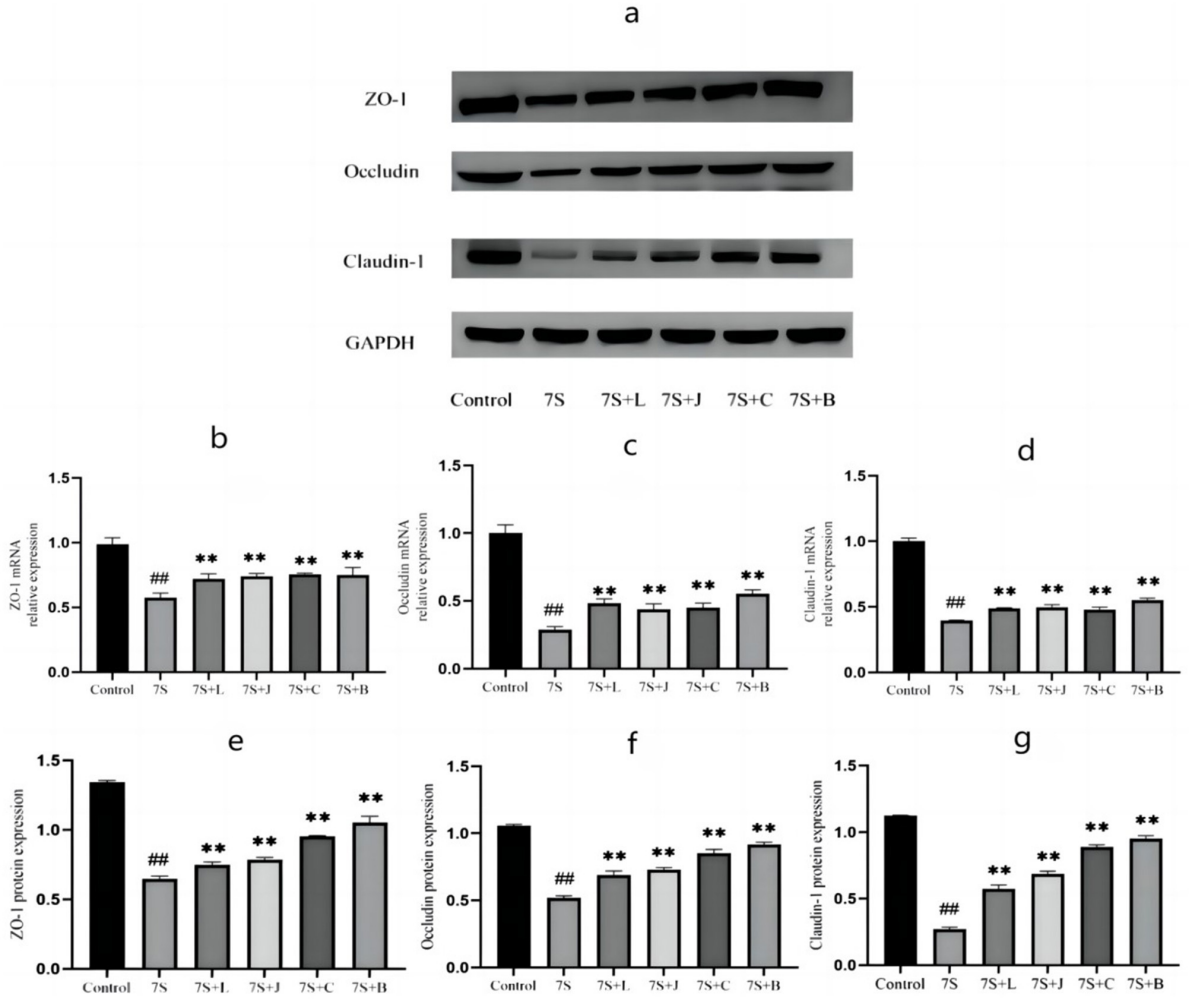
Effect of four traditional Chinese medicine monomers on tight Junction mRNA and protein expression in IPEC-J2 cells caused by soybean 7S globulin. **(A)** The western blot results of ZO-1, Occludin, Claudin-1, and GAPH protein expression. **(B–D)** The changes of ZO-1, Occludin, and Claudin-1 mRNA expression levels. **(E–G)** The quantification of ZO-1, Occludin, and Claudin-1 protein expression. 7S+L means 7S+40 μg/mL of forsythia A; 7S+J means 7S+0.08 μg/mL of curcumin; 7S+C means 7S+50 μg/mL of eleu-theroside E; 7S+B means 7S+50 μg/mL of saponin B4. Data are presented with the means ± standard deviation. **(Compared with the 7S group) or ^*##*^(Compared with the control group) means *P* < 0.01.

Based on above-mentioned results, curcumin at 0.08 μg/mL, eleutheroside E at 50 μg/mL, saponin B4 at 50 μg/mL, and forsythia A at 40 μg/mL exhibited the most pronounced protective effects. Thus, we use 0.08 μg/mL curcumin, 50 μg/mL eleutheroside E, 50 μg/mL saponin B4, and 40 μg/mL forsythia A for next experiment. As shown in Figure 6, the expression of occludin, claudin-1, and ZO-1 in IPEC-J2 cells treated with soybean 7S globulin. Compared to the 7S group, the mRNA (Figures 6B–D) and protein (Figures 6E–G) expression levels of ZO-1, occludin, and claudin-1 were elevated (*P* < 0.01) in response to treatment with the four TCM monomers. Among these, saponin B4 at 50 μg/mL showed the highest relative mRNA and protein expression in IPEC-J2 cells. These findings strongly suggest that the four TCM monomers play a crucial role in protecting the intestinal barrier and saponin B4 at 50 μg/mL has strongest protective effect.

### Effect of saponin B4 on the protein expression of the Rho/ROCK signaling pathway in IPEC-J2 cells induced by soybean 7S globulin

**Figure 7:**
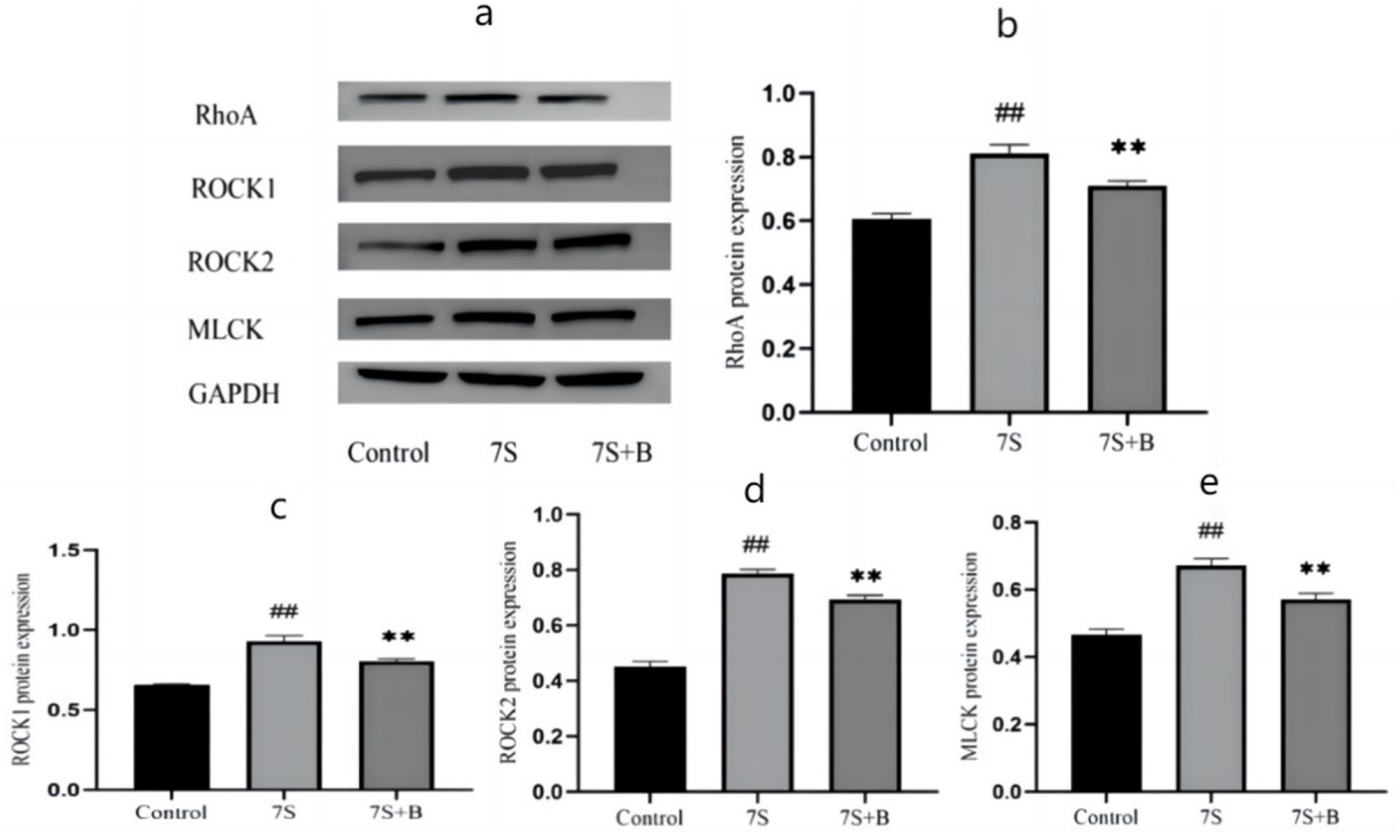
Effect of saponin B4 on the expression of the Rho/ROCK signaling path-way-related proteins in 7S IPEC-J2 cells. **(A)** The western blot results of RhoA, ROCK1, ROCK2, MLCK, and GAPDH protein expression. The quantification of RhoA **(B)**, ROCK1 **(C)**, ROCK2 **(D)**, and MLCK **(E)** protein expression. 7S+B means 7S+50 μg/mL of saponin B4. Data are presented with the means ± standard deviation. **(Compared with the 7S group) or ^*##*^(Compared with the control group) means *P* < 0.01.

Among the four TCM monomers, saponin B4 exhibited the strongest preventive and therapeutic effects. To explore whether saponin B4 alleviates the damage caused by soybean 7S globulin in IPEC-J2 cells via the Rho/ROCK signaling pathway, we conducted further investigation. Compared to the control group, treatment with soybean 7S globulin notably increased (*P* < 0.01) the protein expression of RhoA, ROCK1, ROCK2, and MLCK in the Rho/ROCK signaling pathway. However, co-treatment with 50 μg/mL of saponin B4 and soybean 7S globulin resulted in a marked decrease in the expression of RhoA, ROCK1, ROCK2, and MLCK (*P* < 0.01) compared to the 7S group (Figure 7).

## Discussion

It is well known that soybean protein is a primary source of animal feed, with soybean 7S globulin being the most significant allergen responsible for diarrhea in diets containing soybean protein. Numerous studies have demonstrated the preventive and therapeutic effects of TCMs, such as curcumin, eleutheroside E, saponin B4, and forsythia A, in improving intestinal damage in human and animals. Wang et al. (22) proved that curcumin have antiviral against Porcine deltacoronavirus (PDCoV) and preventing diarrhea caused by PDCoV. Eleutheroside E exhibits immunocompetence, antioxidant, and anti-inflammatory activity, and can protects the Lipopolysaccharides (LPS)-induced increase in permeability of IPEC-J2, potentially by expressing high levels of TJ proteins and inhibiting the increase of inflammatory cytokines (23). In a mouse colitis model, saponin B4 has also been shown to alleviate intestinal damage and treat colitis by regulating macrophage function, inhibiting (nuclear factor kappa-B) NF-κB signaling pathway (19, 24). Forsythia A also been proved to regulated intestinal homeostasis in mice with Ulcerative colitis by improving the gut microbiota and enhancing the intestinal metabolites short-chain fatty acid (19). However, it remains unclear whether these TCM monomers can prevent diarrhea specifically caused by soybean 7S globulin, and the mechanisms underlying their preventive effects have been scarcely reported. In this study, IPEC-J2 cells were used as the experimental model and treated with soybean 7S globulin along with the four TCM monomers (curcumin, eleutheroside E, saponin B4, and forsythia A). The results indicated that all four monomers improved cell viability, suggesting that curcumin, eleutheroside E, saponin B4, and forsythia A exert protective effects on IPEC-J2 cells damaged by soybean 7S globulin.

To further investigate the specific mechanisms of the four TCM monomers alleviate cellular injury caused by soybean 7S globulin, we examined epithelial permeability, integrity, inflammation response, and TJ protein expression in IPEC-J2 cells. TEER and permeability of sodium fluorescein is used as an indicator of epithelial barrier function, reflecting changes in cell monolayer integrity and permeability (25). The current study demonstrated that soybean 7S globulin significantly reduced TEER, while curcumin, eleutheroside E, saponin B4, and forsythia A effectively increased TEER and decreased the permeability of sodium fluorescein. LDH, ALP, DAO, and IFABP1 are key intracellular enzymes or proteins in intestinal epithelial cells, commonly used as indicators of intestinal mucosal damage (26). Our results showed that curcumin, eleutheroside E, saponin B4, and forsythia A significantly diminished the levels of these markers, indicating that they can alleviate the damage to IPEC-J2 cell integrity caused by soybean 7S globulin. Liu et al. (27) reported similar findings in BGC-823 cells treated with curcumin, showing that curcumin modulated cellular permeability, which is consistent with our results. Inflammation is a localized tissue response to damage, and when soybean 7S globulin enters the intestinal tract and triggers an allergic reaction, it also activates an inflammatory response, promoting the release of pro-inflammatory cytokines. Our studies indicated that curcumin, eleutheroside E, saponin B4, and forsythia A alleviated soybean 7S globulin-induced inflammation, as evidenced by reduced levels of IL-1β, IL-6, and TNF-α, as well as increased levels of IL-4 and IL-10. These findings are supported by previous studies, where Xia et al. (28) and Fan (29) also demonstrated that TCM monomers could reduce IL-6 and TNF-α expression (*P* < 0.05) and enhance IL-10 expression (*P* < 0.05).

TJs in intestinal epithelial cells form a crucial structural component of the intestinal mechanical barrier, primarily consisting of the ZO, occludin, and claudin protein families. When the expression of these proteins are reduced, TJs are disrupted, leading to damage to the intestinal mucosal barrier and compromised protection of the intestinal tract. Research has shown that traditional medicines can mediate the expression of TJ proteins to preserve intestinal mucosal function. Xun et al. (30) found that treatment with 2 mg/kg of curcumin significantly increased the mRNA expression of ZO-1 and occludin (*P* < 0.05). Similarly, Li (31) confirmed that saponin B4 enhanced the expression of occludin, claudin-1, and ZO-1 mRNA (*P* < 0.01). These findings align with the results of our study, where TCM monomers effectively alleviated the disruption of TJs caused by soybean 7S globulin (16, 32). Moreover, our experiment was the first to confirm that Forsythia A can significantly increase the mRNA expression levels of claudin-3, occludin, and ZO-1, thus contributing to the protection of the intestinal mechanical barrier.

The intestinal mechanical barrier, particularly the integrity of its TJs, is the first line of defense in the digestive system and plays a crucial role in maintaining the health of the pig population. The Rho/ROCK signaling pathway has been confirmed to regulate the intestinal mechanical barrier (33). Research indicates that Rho undergoes conformational changes to RhoA by binding with GTP, and ROCK acts as a downstream effector molecule of RhoA. Upon activation, these molecules sequentially phosphorylate the MLC pathway, inducing myosin contraction and disrupting TJs (34). This process alters the basic structure of junction proteins, ultimately leading to damage to the intestinal mechanical barrier. Wu et al. (35) found that treatment with 5 mg/mL of soybean 7S globulin caused barrier damage in IPEC-J2 cells, with significantly increased expression of MLCK, ROCK1, and ROCK2 (*P* < 0.01), suggesting that soybean 7S globulin can damage TJs via the Rho/ROCK signaling pathway (36, 37). Additionally, reports have shown that resveratrol and catalpol can modulate the Rho/ROCK signaling pathway. In our experiments, we confirmed that 50 μg/mL of saponin B4 notably reduced the expression levels of RhoA, ROCK1, ROCK2, and MLCK (*P* < 0.01), thereby affecting the Rho/ROCK signaling pathway and protecting TJs. These findings suggest that the Rho/ROCK signaling pathway may be a target for TCMs to ameliorate damage to the intestinal mechanical barrier in epithelial cells.

In conclusion, our results demonstrated that curcumin (0.02, 0.04, and 0.08 μg/mL), eleutheroside E (25 and 50 μg/mL), saponin B4 (12.5, 25, and 50 μg/mL), and forsythia A (10 and 40 μg/mL) could alleviate cell death, permeability damage, integrity loss, inflammatory response, and mechanical barrier damage in IPEC-J2 cells induced by soybean 7S globulin. Among these, curcumin at 0.08 μg/mL, eleutheroside E at 50 μg/mL, saponin B4 at 50 μg/mL, and forsythia A at 40 μg/mL exhibited the most pronounced protective effects. The Rho/ROCK signaling pathway may be one of the key mechanisms by which these TCM monomers protect against mechanical barrier damage in intestinal epithelial cells.

## Data Availability

The original contributions presented in the study are included in the article/Supplementary material, further inquiries can be directed to the corresponding authors.

## Supplementary material

Data Sheet 1 The original image of the protein strip demonstrating the mechanical barrier and Rho/ROCK signaling pathway related protein expression.
