# Supplementary figures and images for: Effect of four traditional Chinese medicine monomers on mechanical barrier damage and inflammation response of IPEC-J2 cells caused by soybean 7S globulin
Source: Front Vet Sci. 2025 Mar 7;12:1548866. doi: 10.3389/fvets.2025.1548866 (PMC11925905; doi:10.3389/fvets.2025.1548866)

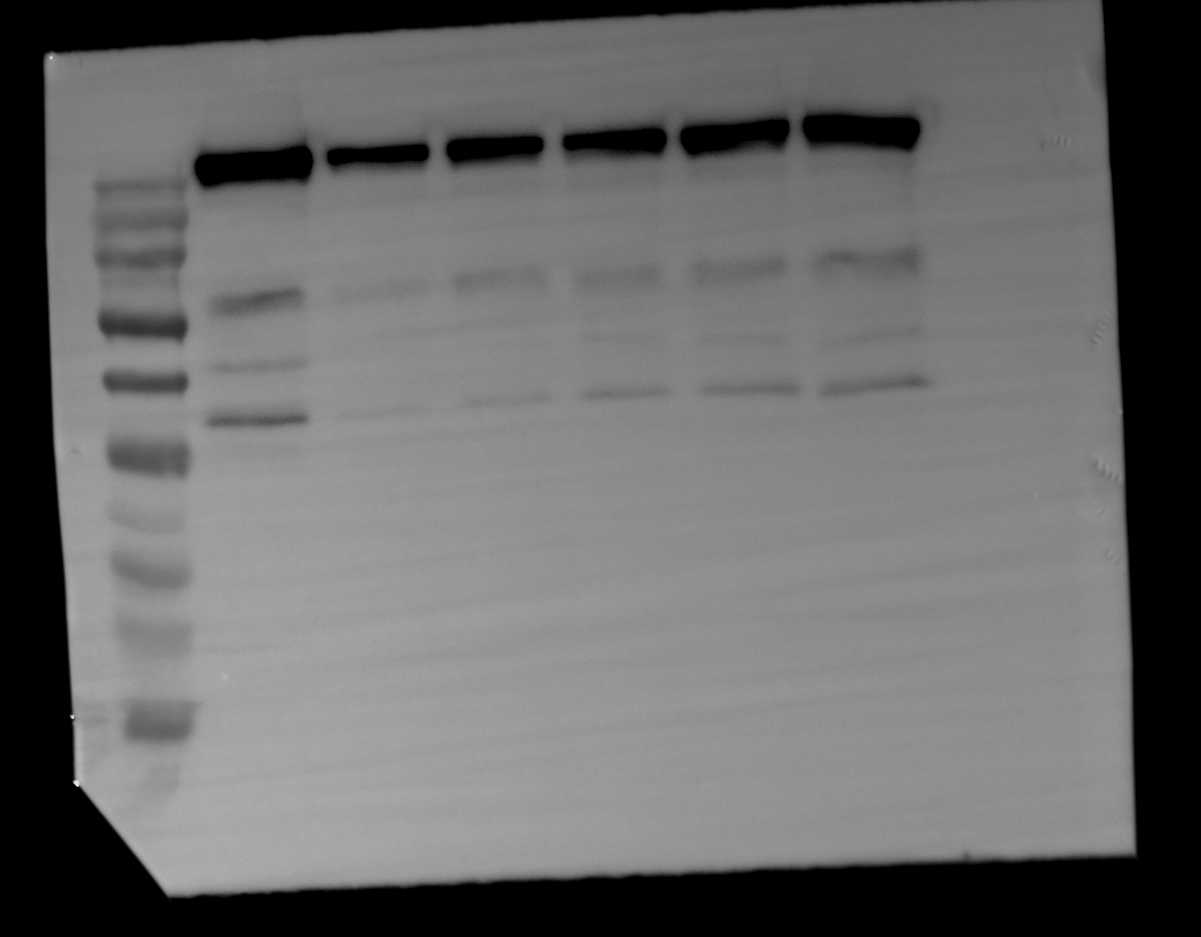

Supplement: Data Sheet 1 — The original image of the protein strip demonstrating the mechanical barrier and Rho/ROCK signaling pathway related protein expression. [file Data_Sheet_1.zip › ZO-1 一.tif]

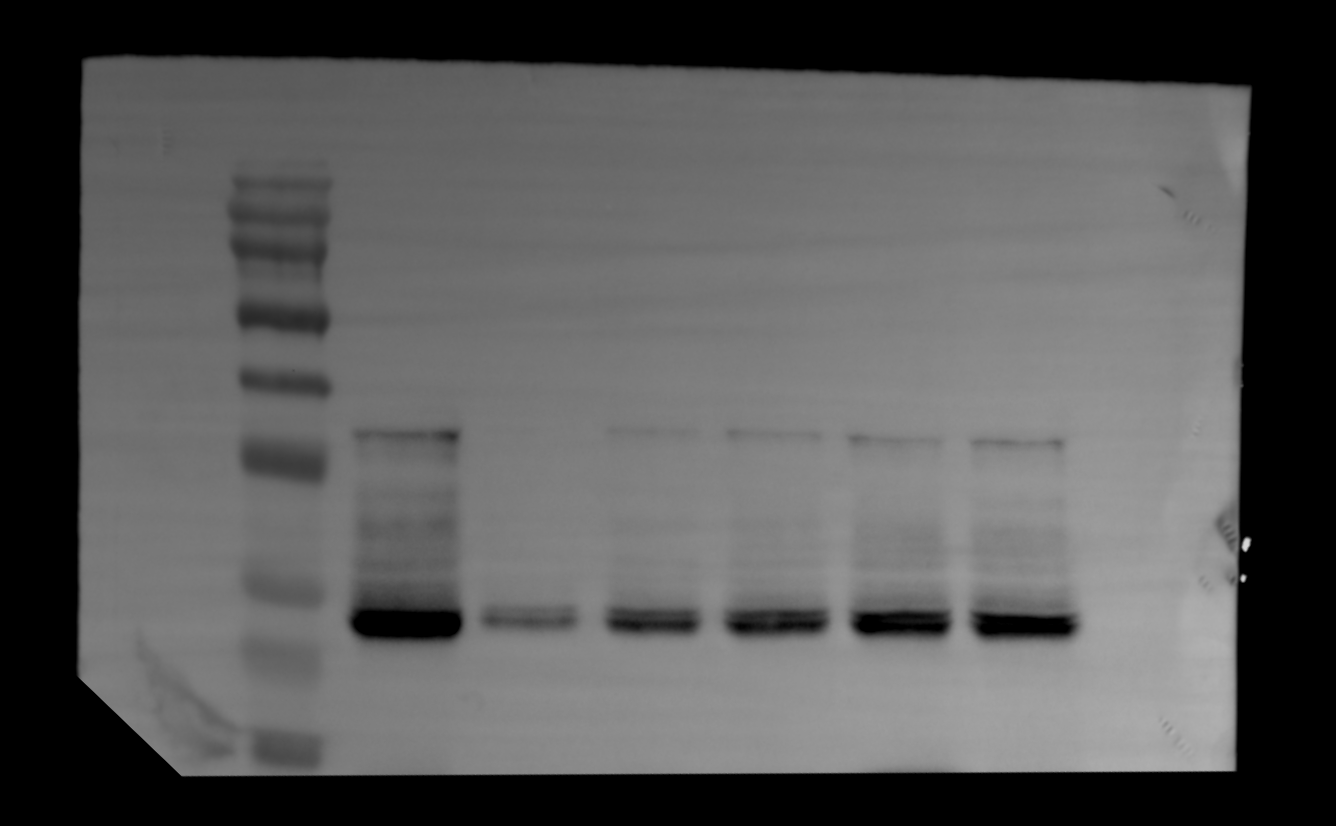

Supplement: Data Sheet 1 — The original image of the protein strip demonstrating the mechanical barrier and Rho/ROCK signaling pathway related protein expression. [file Data_Sheet_1.zip › claudin1 一.tif]

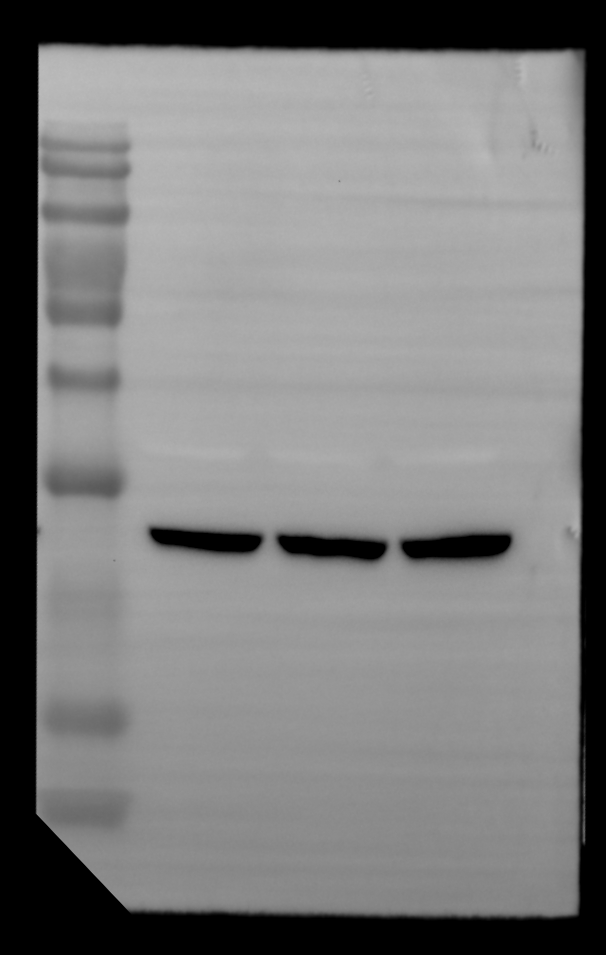

Supplement: Data Sheet 1 — The original image of the protein strip demonstrating the mechanical barrier and Rho/ROCK signaling pathway related protein expression. [file Data_Sheet_1.zip › GAPDH 二.tif]

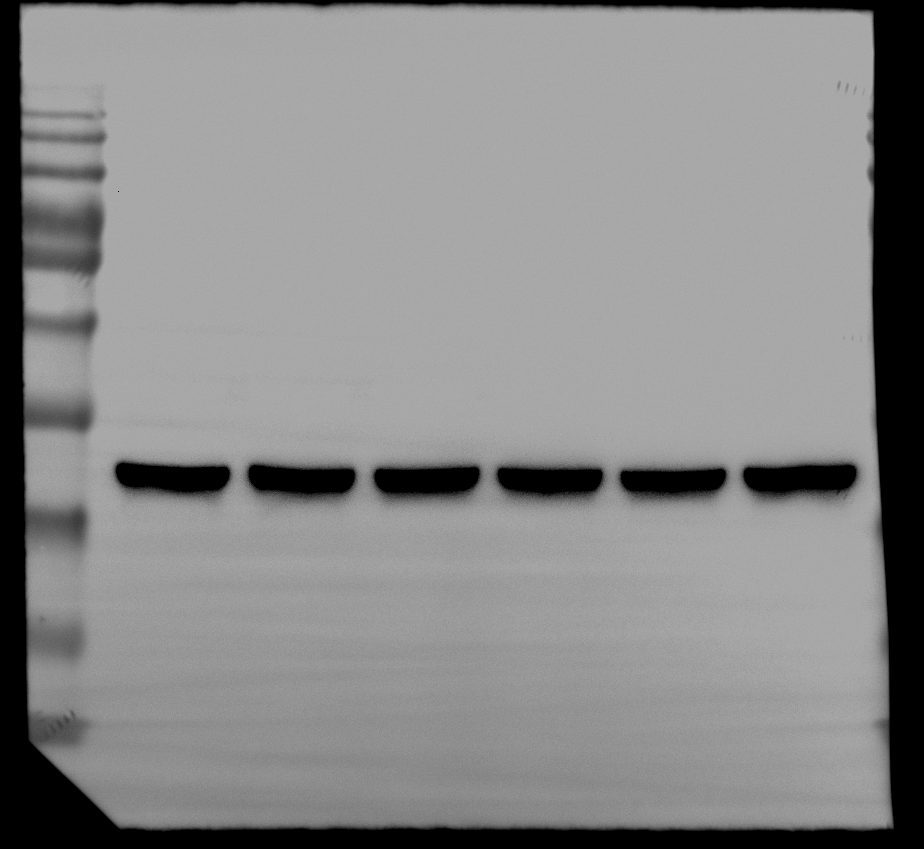

Supplement: Data Sheet 1 — The original image of the protein strip demonstrating the mechanical barrier and Rho/ROCK signaling pathway related protein expression. [file Data_Sheet_1.zip › GAPDH 一.tif]

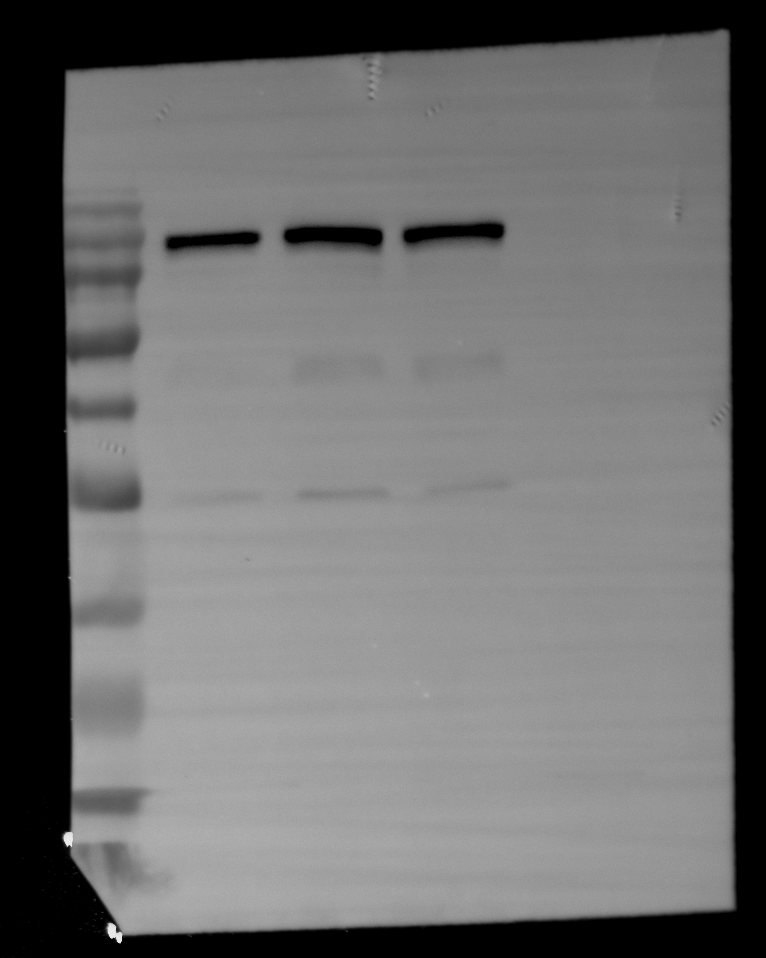

Supplement: Data Sheet 1 — The original image of the protein strip demonstrating the mechanical barrier and Rho/ROCK signaling pathway related protein expression. [file Data_Sheet_1.zip › MLCK 二.tif]

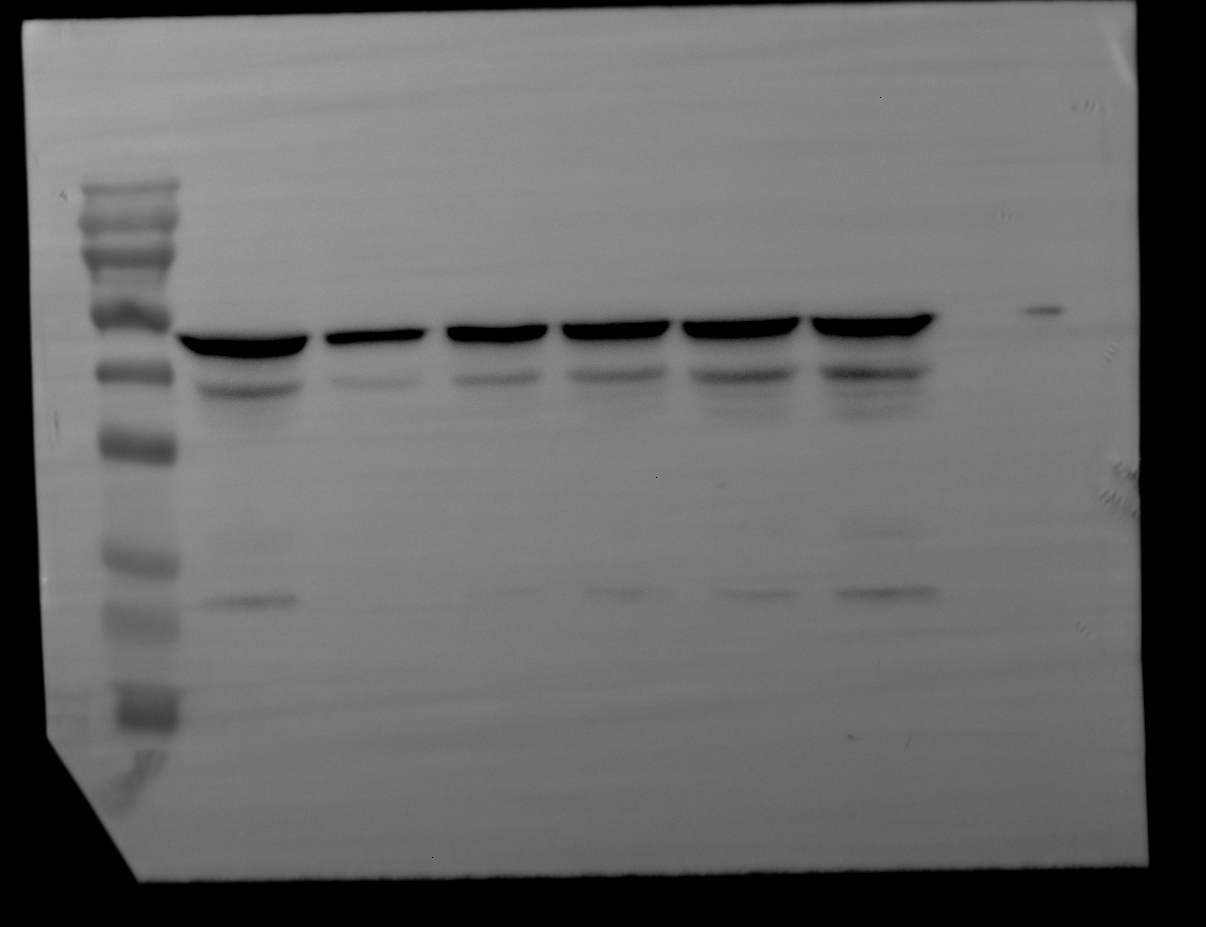

Supplement: Data Sheet 1 — The original image of the protein strip demonstrating the mechanical barrier and Rho/ROCK signaling pathway related protein expression. [file Data_Sheet_1.zip › Occludin 一.tif]

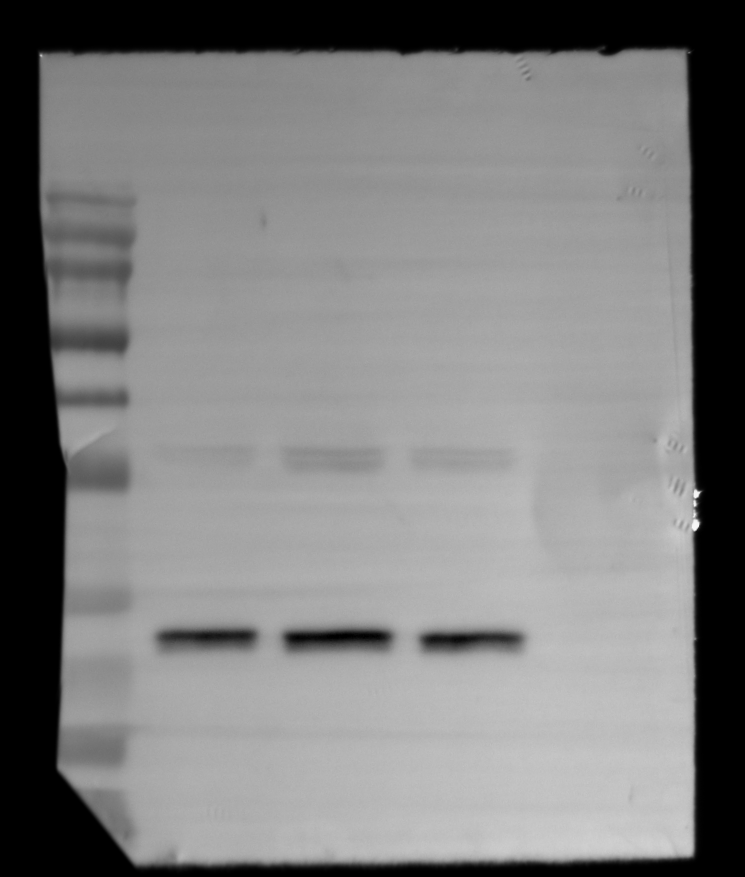

Supplement: Data Sheet 1 — The original image of the protein strip demonstrating the mechanical barrier and Rho/ROCK signaling pathway related protein expression. [file Data_Sheet_1.zip › RhoA 二.tif]

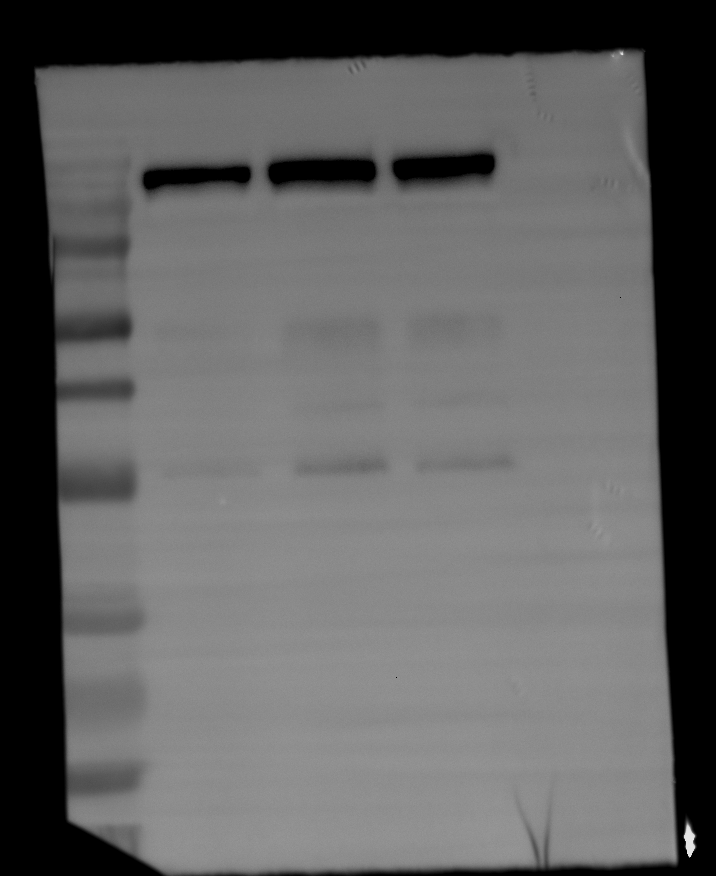

Supplement: Data Sheet 1 — The original image of the protein strip demonstrating the mechanical barrier and Rho/ROCK signaling pathway related protein expression. [file Data_Sheet_1.zip › ROCK1 二.tif]

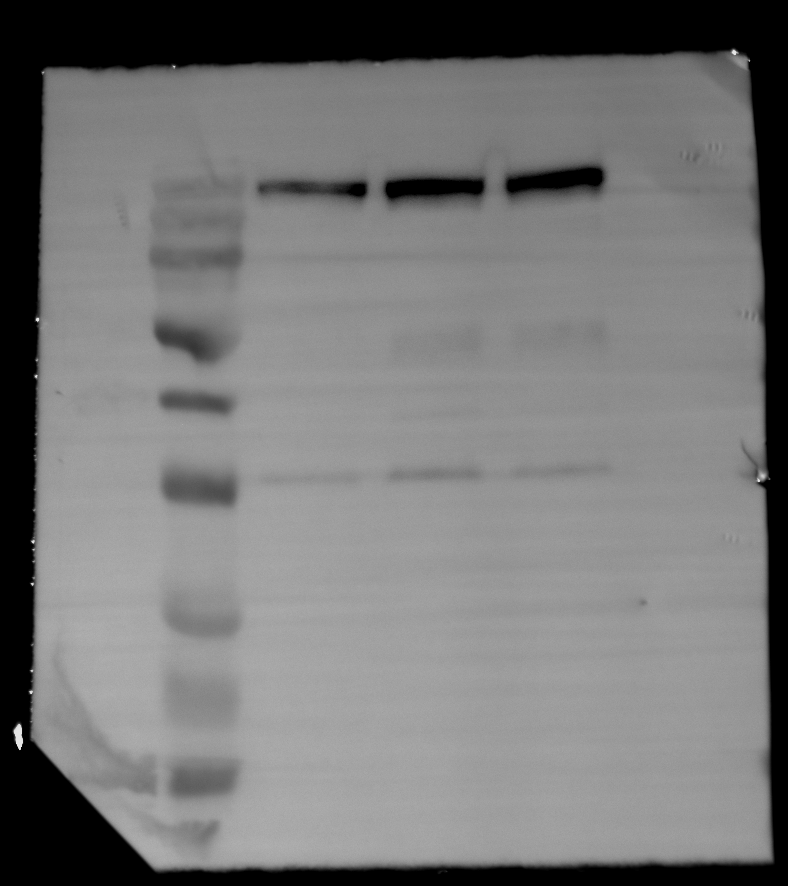

Supplement: Data Sheet 1 — The original image of the protein strip demonstrating the mechanical barrier and Rho/ROCK signaling pathway related protein expression. [file Data_Sheet_1.zip › ROCK2 二.tif]
